# Mitochondrial Transplantation Increases Dermal Fibroblast Proliferation and Accelerates Acute Wound Closure

**DOI:** 10.1111/wrr.70192

**Published:** 2026-07-24

**Authors:** Ikkei Takashimizu, Miho Noguchi, Shunsuke Yuzuriha

**Affiliations:** ^1^ Department of Plastic and Reconstructive Surgery Shinshu University School of Medicine Matsumoto Japan

**Keywords:** cell proliferation, isolated mitochondria, mitochondrial transplantation, wound healing

## Abstract

The detrimental effects of dysfunctional mitochondria on cells and tissues have been shown to improve by healthy mitochondria transplantation. However, the feasibility of intercellular mitochondrial transfer for wound healing remains uncertain. This study examined whether healthy mitochondria transplantation promoted the healing of acute wounds. Isolated mitochondria derived from primary cultured normal human dermal fibroblasts were administered to primary cells of the same lineage, with confirmed cell uptake. Wound closure rate in in vitro scratch wound healing assays increased in a dose‐dependent manner and was attenuated by treatment with the mitochondrial function inhibitor antimycin A. Gene expression analysis revealed significant increases in the cell proliferation markers *MKI67* and *PCNA*, although no consistent changes in migration‐related genes were observed. Cell proliferation rate increased to a maximum average of 1.4‐fold after isolated mitochondrial administration, which was attenuated by antimycin A treatment. To verify the in vivo effects of isolated mitochondrial transplantation, mitochondria were obtained from mouse livers, and wound closure rate was analysed using a mouse wound healing model. The number of days to wound closure was significantly shortened in a dose‐dependent manner, and this gain was attenuated by antimycin A. Our findings demonstrate that mitochondrial transplantation accelerates acute wound closure and enhances fibroblast proliferative activity. The observed increase in cell proliferation may contribute, at least in part, to the beneficial effects of mitochondrial transplantation on wound healing. These findings provide insight into the therapeutic potential of mitochondrial transplantation as a novel strategy for wound repair.

## 1 Introduction

In recent years, cell‐based therapies such as regenerative medicine using autologous cultured epidermis [1, 2], somatic stem cells [3], embryonic stem cells [4, 5], and induced pluripotent stem cells [6, 7], as well as chimeric antigen receptor T‐cell therapy [8, 9] that introduce genes into harvested patient cells and return them to the body, have been performed in the clinical setting. However, treatments focusing on intracellular organelles often remain in the research stage due to insufficient functional analysis.

Mitochondria are crucial intracellular organelles involved in energy production. These cells cannot self‐repair from functional abnormalities, which may affect cells, tissues, and the organism as a whole. Mitochondrial impairment can lead to a wide range of diseases, including diabetes [10, 11, 12], cardiovascular diseases [13], Parkinson's disease [14, 15], dementia [16, 17], mood disorders [18], malignant tumours [19, 20], and lifestyle‐related diseases [21]. On the other hand, the transplantation of healthy mitochondria into cells with dysfunctional mitochondria may restore cellular function [22, 23, 24, 25]. This has led to the proposed phenomenon of mitochondrial transfer, whereby mitochondria are exchanged among cells. As such, supplemented mitochondria from external sources may compensate for the dysfunction of damaged mitochondria. Recent studies have described that mitochondrial transplantation improved both cellular and tissue function in myocardial ischemia–reperfusion injury [26], acute lung injury [27], nerve injury [28], Parkinson's disease [29], acute myelogenous leukaemia [30], non‐alcoholic fatty liver disease [31], and mitochondrial disease [32]. These findings indicate that the transplantation of isolated mitochondria (iMt) into damaged cells or tissues has therapeutic potential.

During the wound healing process, cells require an enormous amount of energy for the growth and synthesis of cellular materials, most of which depend on the energy produced by mitochondria [33, 34, 35]. Therefore, energy supply may be irreversibly reduced by mitochondrial dysfunction, resulting in prolonged wound healing. Indeed, the intracellular administration of adenosine triphosphate (ATP), an intracellular energy currency, was found to accelerate wound healing [36]. The present study evaluated the effects of transplanted functional mitochondria isolated from healthy cells into cells with acute wounds both in vitro and in vivo.

## 2 Materials and Methods

All experiments were carried out in accordance with the Regulations for the Handling of Animal Experiments. The protocols were approved by the Animal Care and Use Committee of Shinshu University (approval no. 024039). In addition, all methods were conducted in adherence to the ARRIVE guidelines.

### 2.1 Cells and Animals

Normal human dermal fibroblasts (NHDF; CC‐2511) were purchased from Lonza Walkersville Inc. (MD, USA). Cell culture medium was composed of D‐MEM (041‐29775; Fujifilm Wako Pure Chemical Corp., Osaka, Japan) supplemented with 10% fetal bovine serum (Gibco, Thermo Fisher Scientific, MA, USA) and 1% penicillin/streptomycin (168‐23191; Fujifilm Wako Pure Chemical Corp.). The cells were incubated at 37°C under 5% CO_2_. For mitochondria isolation, cells were cultured in φ150 mm culture dishes. Cells at 80%–100% confluence were collected by trypsinization and used for ensuing experiments.

Male C57BL/6NJ mice were purchased at 5 weeks of age from Jackson Laboratory Japan Inc. (Yokohama, Japan), housed under specific pathogen‐free conditions in the Division of Animal Research, Research Center for Advanced Science and Technology, Shinshu University, and used for experiments at 6 weeks of age. The animals were fasted from the day before mitochondrial isolation and used for subsequent analyses.

### 2.2 Anaesthesia

Three anaesthetic mixtures of midazolam (Dormicum, Maruishi Pharmaceutical Co. Ltd., Osaka, Japan), butorphanol tartrate (Vetorphale, Meiji Animal Health Co. Ltd., Kumamoto, Japan), and medetomidine hydrochloride (Domitor, Nippon Zenyaku Kogyo Co. Ltd., Fukushima, Japan) were used on mice at doses of 4 mg/kg body weight (BW), 5 mg/kg BW, and 0.3 mg/kg BW, respectively. Prior to liver extraction for mitochondrial isolation, an intraperitoneal overdose of the three anaesthetic mixtures five times larger than the ordinary dose was administered.

In the wound healing assay using mice, a mixture of the three anaesthetics was administered subcutaneously (0.05 mL/10 g BW) to induce general anaesthesia. Following treatment, atipamezole hydrochloride (Antisedan, Nippon Zenyaku Kogyo Co.) was injected subcutaneously at a dose of 0.03 mg/10 g BW to awaken the mice. The mice were returned to their cages when fully awake and maintained in an appropriate environment.

### 2.3 Immunocytochemistry

Cultured cells were fixed with 4% paraformaldehyde in phosphate buffer saline (PBS) for 10 min at room temperature and permeabilized with 0.1% Triton X‐100 (Fujifilm Wako Pure Chemical Corp.) for 10 min at room temperature. PBS supplemented with 3% normal goat serum (S‐1000; Vector Laboratories Inc., Burlingame, CA, USA) was used as a protein‐blocking solution. The cells were incubated overnight at 4°C with anti‐Tomm40 (18409‐1‐AP at 1:200 dilution; Proteintech Group Inc., IL, USA) as a mitochondria‐specific marker. Alexa Fluor 594 goat anti‐rabbit IgG (H + L) antibody (A11037 at 1:1000 dilution; Invitrogen, Thermo Fisher Scientific, MA, USA) was adopted as a secondary antibody. Nuclei were stained with DAPI (S96369; Invitrogen, Thermo Fisher Scientific). Immunofluorescence images were visualised and recorded using a BX53 fluorescence microscope (Olympus, Tokyo, Japan).

### 2.4 Isolation of Mitochondria From Cultured Cells and Mouse Livers

We adopted a modified previously reported method to isolate mitochondria from NHDF [11, 12, 13]. Briefly, cells were homogenised in homogenization buffer (30 mM Tris‐HCl pH 7.4, 225 mM mannitol, 75 mM sucrose, and 0.1 mM EGTA) by 20 strokes gently with a 27‐gauge needle (NN‐2719S; Terumo Corp., Tokyo, Japan) and 1 mL syringe (SS‐01T; Terumo Corp.) on ice. After centrifuging the cell lysate at 600 × g for 10 min at 4°C, the supernatant was collected in a separate tube. The pellet was resuspended in homogenization buffer and stroked 10 times again with the same needle and syringe. Following centrifugation under the same conditions, the supernatant was transferred to the separate tube. After two rounds of discarding the supernatant following centrifugation at 8000 × g for 10 min at 4°C, the pellet was washed in fractionation buffer (30 mM Tris‐HCl pH 7.4, 225 mM mannitol, and 75 mM sucrose), and the iMt fraction pellet was resuspended in mitochondria resuspension buffer (5 mM HEPES‐KOH pH 7.4, 250 mM mannitol, and 0.5 mM EGTA) for experiments. The total protein concentration in each fraction was determined using a Bradford Protein Assay kit (T9310A; Takara Bio Inc., Shiga, Japan) according to the manufacturer's instructions.

To isolate healthy mitochondria from C57BL/6NJ mice, 6‐week‐old animals were anaesthetised with a mixture of the three anaesthetics described above. After laparotomy, cooled PBS was infused through the portal vein to flush the blood from the liver. Then, the organ was removed and lightly washed with cooled washing buffer (30 mM Tris‐HCl pH 7.4, 225 mM mannitol, and 75 mM sucrose), cut into 1 mm cubes with a razor blade, and homogenised with cooled homogenization buffer for liver (30 mM Tris‐HCl pH 7.4, 225 mM mannitol, 75 mM sucrose, 0.5% BSA, and 0.5 mM EGTA). The sample was then centrifuged at 700 × g for 10 min at 4°C, and the supernatant was transferred to a separate tube. This procedure was repeated, followed by centrifugation at 8000 × g for 10 min at 4°C. The supernatant was removed, and the pellet was resuspended in fractionation buffer for liver (30 mM Tris‐HCl pH 7.4, 225 mM D‐mannitol, 75 mM sucrose, and 0.5% BSA). After centrifugation at 8000 × g for 10 min at 4°C, the supernatant was removed, and the precipitate was washed with fresh fractionation buffer for liver. This process was repeated, and the remaining precipitate was resuspended in mitochondria resuspension buffer for liver (5 mM HEPES‐KOH pH 7.4, 250 mM D‐mannitol, and 0.5 mM EGTA) to obtain the mitochondrial fraction. The protein concentration of each fraction was determined using a Bradford Protein Assay Kit, as described above.

### 2.5 Western Blot Analysis

Samples were taken from each mitochondrial fraction obtained during the isolation process for cultured cells and mouse livers. These samples included whole cells, crushed sediment, nucleus‐free supernatant, and both the supernatant and precipitate from the mitochondrial fraction. Based on the protein concentration of each measured sample, 0.2 μg/μL samples were prepared with mitochondria resuspension buffer and SDS sample buffer (0.125 M Tris‐HCl buffer pH 6.8, 10% 2‐mercaptoethanol, 4% SDS, 10% sucrose, and 0.01% bromophenol blue) and heat‐treated at 95°C for 3 min. TGX FastCast gel (1610173; Bio‐Rad Laboratories Inc., CA, USA) was prepared according to the manufacturer's instructions, and SDS‐PAGE was performed using Mini‐PROTEAN Tetra Cells (10007296; Bio‐Rad Laboratories Inc.). Electrophoresis was conducted at a constant voltage of 200 V for 36 min. Afterwards, transfer treatment was performed on Immun‐Blot PVDF Membranes for Protein Blotting (1620177; Bio‐Rad Laboratories) under a constant voltage of 100 V for 40 min, and then blocking was performed with 2% skim milk (190‐12865; Fujifilm Wako Pure Chemical Corp.)/0.5% Tween‐20 (166‐21213; Fujifilm Wako Pure Chemical Corp.)/PBS solution after washing. Each primary antibody was diluted with the same solution and subjected to shaking at 4°C for 12 h. The conditions for each primary antibody used were as follows: anti‐Tomm40 antibody (1:5000 dilution), anti‐COX IV antibody (ab33985 at 1:2000 dilution; Abcam plc, Cambridgeshire, GB), anti‐cytochrome C antibody (ab13575 at 1:1000 solution; Abcam plc), anti‐calnexin antibody (10427‐2‐AP at 1:10000 dilution; Proteintech Group Inc), and anti‐histon‐H3 antibody (17168‐1‐AP at 1:5000 dilution; Proteintech Group Inc). The secondary antibodies were treated with anti‐mouse/rabbit IgG, HRP‐linked antibody (no. 7076/7074 at 1:2000 dilution; Cell Signalling Technology Inc., MA, USA) for 1 h at room temperature with shaking. Detection was performed with Amersham ELC Prime Western Blotting Detection Reagent (RPN2232; GE Healthcare, IL, USA) and exposure using the ChemiDoc Touch imaging system (Bio‐Rad Laboratories).

### 2.6 Transmission Electron Microscopic Observation

The iMt derived from NHDF and mouse livers were centrifuged to form pellets, fixed with 2.5% glutaraldehyde overnight at 4°C, and fixed again with 1% osmium tetroxide for 1 h at 4°C. After dehydration in graded ethanol, replacement with propylene oxide, and embedding in epoxy resin, the samples were sectioned at approximately 1 μm thickness. The specimens were trimmed, and then ultra‐thin sections 60–100 nm thick were obtained with a diamond knife. Electron staining and vacuum evaporation with carbon were conducted next. All images were visualised and recorded using a JEM‐1400 transmission electron microscope (JEOL Ltd., Tokyo, Japan).

### 2.7 Measurement of Oxygen Consumption Rate (OCR)

OCR was measured with an Extracellular O_2_ Consumption Assay (ab197243; Abcam plc) for the functional assessment of iMt according to the manufacturer's directions. Briefly, after isolating mitochondria from NHDF cultured to 80%–100% confluence and from mouse livers, we added OCR reagent, substrates, adenosine diphosphate and/or antimycin A (A5378; LKT Laboratories Inc., MN, USA), which was an inhibitor of the enzyme complex III of the electron transport chain in mitochondria. The specific reaction fluorescence signal was detected with a SpectraMax iD5 plate reader (Molecular Devices, CA, USA).

### 2.8 Mitochondrial Uptake Assay

To evaluate mitochondrial uptake by NHDF, isolated mitochondria were fluorescently labelled using MitoGreen (Promokine, PK‐CA707‐70054) according to the manufacturer's instructions. Briefly, isolated mitochondria were incubated with MitoGreen at 37°C for 1 h. Following labelling, mitochondria were collected by centrifugation and purified using the same isolation procedure described above to remove excess dye. The labelled mitochondria were administered to NHDF and incubated overnight at 37°C under 5% CO_2_ conditions. The following day, cells were either subjected to fluorescence imaging or flow cytometric analysis. For fluorescence imaging, the culture medium was replaced with fresh medium, nuclei were counterstained with Hoechst 33342, and cells were observed using a TCS SP8 confocal laser scanning microscope (Leica Microsystems, Wetzlar, Germany). Representative images were obtained to confirm intracellular localization of transplanted mitochondria. For flow cytometric analysis, cells were detached by trypsinization, washed with phosphate‐buffered saline, and analysed using a BD FACSCelesta flow cytometer (BD Biosciences, NJ, USA). Untreated NHDF were used as negative controls for gating, and the percentage of MitoGreen‐positive cells was determined.

For in vivo uptake analysis, isolated mitochondria were fluorescently labelled with MitoGreen as described above. Labelled mitochondria were administered to wounds immediately after treatment. Control wounds received mitochondria resuspension buffer alone. Twenty‐four hours later, wound tissues were harvested, embedded in OCT compound, and cryosectioned. Tissue sections were counterstained with DAPI and examined using a LSM 880 confocal laser scanning microscope (Carl Zeiss, Oberkochen, Germany). Representative fluorescence images were obtained to evaluate the distribution of MitoGreen‐derived fluorescence signals within wound tissues. Details of the wound model and treatment protocol are described below.

### 2.9 Scratch Wound Healing Assay

The day prior to the wound healing assay, NHDF were subcultured to a new gelatin‐coated 6‐well plate at a density of 2.5 × 10^5^/well and incubated at 37°C under 5% CO_2_. Approximately 1 mm wide wounds were made by scratching the surface of confluent NHDF using a micropipette chip. Based on the concentrations of iMt derived from NHDF as determined by the Bradford method, protein concentrations were adjusted by administering 1, 5, and 10 μg of iMt/cm^2^, which were designated as iMt1, iMt5, and iMt10, respectively. Culture medium only was added as the control. For antimycin A experiments, mitochondria were incubated with antimycin A and subsequently washed prior to administration. Control mitochondria underwent the same procedure without antimycin A treatment. The medium was exchanged on the day after the administration of iMt, and then every 2–3 days. For mitochondrial inhibition conditions, each iMt was treated with 1 μM antimycin A and washed before experiments. All cultured cells were incubated in 37°C at 5% CO_2_ conditions and observed periodically using a DMi1 inverted microscope (Leica Microsystems, Wetzlar, Germany). All images were obtained by a Flexacam C1 microscope camera (Leica Microsystems) and analysed using ImageJ version 1.54 g software (NIH, MD, USA) to assess wound healing area.

### 2.10 Gene Expression Analysis

Gene expression analysis was performed using iMt10 samples from NHDF, which demonstrated the greatest efficacy in the scratch wound healing assay. Total RNA was extracted from cells at 12 and 24 h after the administration of iMt10 with a NucleoSpin RNA Plus kit (U0984B; Takara Bio Inc.) according to the manufacturer's instructions. The concentration of the obtained total RNA was measured using a spectrophotometer (NanoDrop One^C^; Thermo Fisher Scientific). Reverse transcription to cDNA was then performed by means of a PrimeScript RT Reagent Kit with gDNA Eraser (RR047Q; Takara Bio Inc.) following the manufacturer's directions. A TaqMan Array 96‐Well FAST Plate Custom Format 32 (4413259; Thermo Fisher Scientific) was used to analyse proliferation (PCNA: Hs00427214_g1, MKI67: Hs00606991_m1, and MAPK1: Hs01046830_m1), migration (RhoA: Hs00357608_m1 and Rac1: Hs01902432_s1), growth factors (FGF2: Hs00266645_m1, VEGFA: Hs00900055_m1, PDGFA: Hs00964426_m1, EGF: Hs01099999_m1, and IGF1: Hs01547656_m1), and membrane receptors (EGFR: Hs01076078_m1 and TGF‐βR: Hs01114253_m1) according to the manufacturer's instructions.

The comparative *C*_*t*_ method (ΔΔ*C*_*t*_ method) was employed to analyse gene expression levels. The *C*_*t*_ values of the target genes were corrected by that of the housekeeping gene GAPDH (Hs99999905_m1) for calculations of the Δ*C*_*t*_ value (Δ*C*_*t*_ = *C*_*t*_target_−*C*_*t*_housekeeping_). The ΔΔ*C*_*t*_ value of each sample was also determined based on the Δ*C*_*t*_ value of the control group (ΔΔ*C*_*t*_ = Δ*C*_*t*_sample_−ΔC_*t*_control_). The final relative expression level of the target genes was obtained by 2^−ΔΔ*Ct*^ power (2^−ΔΔ*Ct*^). Each sample was subjected to three biological and technical repetitions.

### 2.11 Cell Proliferation Assay

NHDF were seeded at a density of 1.0 × 10^4^ cells/well onto gelatin‐coated 6‐well plates and cultured at 37°C under 5% CO_2_. Forty‐eight hours after seeding at the time of the logarithmic growth phase, iMt cultures were treated with either 1 μM antimycin A or vehicles as controls. Vehicle control samples were treated identically except that the solvent used for antimycin A preparation was added without the inhibitor. Untreated control groups received culture medium alone. The medium was changed the next day, and cell counts were determined every 24 h using Countess cell counting chamber slides (C10283; Thermo Fisher Scientific) and a Countess 3 Automated Cell Counter (AMQAX2000; Thermo Fisher Scientific).

### 2.12 Mouse Skin Wound Healing Model and Wound Healing Assay

Six‐week‐old male C57BL/6NJ mice were used. Following anaesthesia, the dorsal hair was shaved, and a full‐thickness skin defect wound of 4 mm in diameter was created. To prevent skin shrinkage, a silicone sheet of 1 mm thickness cut into a doughnut shape was sutured around the wound. Based on the protein concentrations of iMt derived from mouse livers, concentrations were adjusted by administering 33, 66, 100, and 200 μg of iMt/cm^2^, which were designated as iMt33, iMt66, iMt100, and iMt200, respectively. Control wounds received an equivalent volume of mitochondria resuspension buffer without iMt.

Each mitochondrial preparation was either treated with 1 μM antimycin A, washed with mitochondria resuspension buffer, and subjected to the same handling procedure without antimycin A treatment (control mitochondria) prior to administration, and then were allowed to acclimate for 10 min. The mitochondrial suspension was applied topically to the center of the wound bed. Following administration, the suspension spread across the wound surface and covered the entire wound area. A wound dressing material one size larger than the wound was applied for protection and fixed with gauze and tape. Anaesthesia antagonists were administered, and the animals were kept under appropriate conditions after awakening. Wounds were periodically observed, photographed, and analysed using ImageJ software.

### 2.13 Statistics

All statistical analyses were performed using IBM SPSS Statistics version 31 (IBM Corp., Armonk, NY, USA). Data are presented as mean ± standard error of the mean (SEM). For experiments involving repeated measurements over time, including the in vitro scratch wound healing assay, gene expression analysis, cell proliferation assay, and in vivo wound healing assay, two‐way analysis of variance (ANOVA) was used to evaluate the effects of treatment group, time, and treatment‐by‐time interaction. When appropriate, Dunnett's multiple comparisons test was used to compare treatment groups with the corresponding control group, whereas Sidak's multiple comparisons test was used for pairwise comparisons. A *p*‐value of < 0.05 was considered statistically significant.

## 3 Results

**FIGURE 1:**
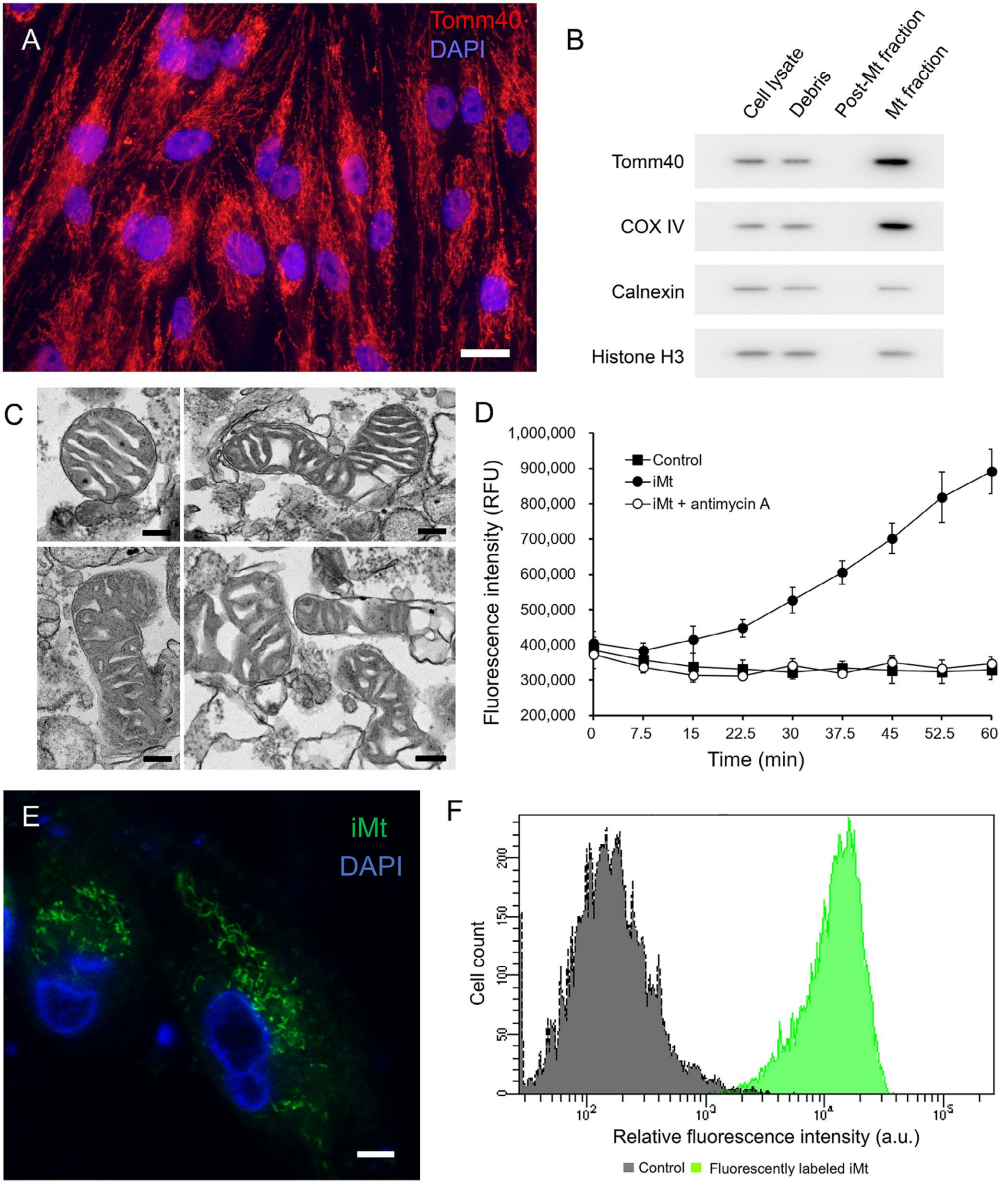
Isolation and functional evaluation of mitochondria derived from normal human dermal fibroblasts (NHDF). (A) Mitochondria present in NHDF. Scale bar = 20 μm. (B) Western blot of each cell fraction obtained from NHDF. (C) Electron microscopy of mitochondria in mitochondrial fraction (Mt fraction). Scale bar = 200 nm. (D) Oxygen consumption rate assay of isolated mitochondria (iMt). Data represent the mean ± standard error of the mean (*n* = 3 for each condition). (E, F) Confocal laser microscopy and flow cytometry of NHDF that have taken up fluorescently labelled iMt (green). Scale bar = 5 μm.

The NHDF used in this study contained abundant mitochondria (Figure 1A). Western blot assays of each fraction after cell disruption confirmed that the mitochondria were isolated and concentrated in the mitochondrial fraction by the mitochondria‐specific anti‐Tomm40 and anti‐COX IV antibodies (Figure 1B). Electron microscopy showed that the iMt were morphologically preserved, with cristae surrounded by a double membrane representing an ultrastructure specific to mitochondria (Figure 1C). We also verified that the iMt retained respiratory activity, an essential indicator of mitochondrial function, by measuring oxygen consumption, which was completely inhibited by the addition of antimycin A (Figure 1D). The above findings demonstrated that functional mitochondria had been isolated. To confirm cellular uptake, the iMt were fluorescently labelled and administered to cultured NHDF. Indeed, fluorescent mitochondria were observable by confocal laser microscopy (Figure 1E), and the fluorescent cell population was confirmed by flow cytometry after detaching and recovering the cells (Figure 1F), proving that the administered iMt were taken up by cells.

**FIGURE 2:**
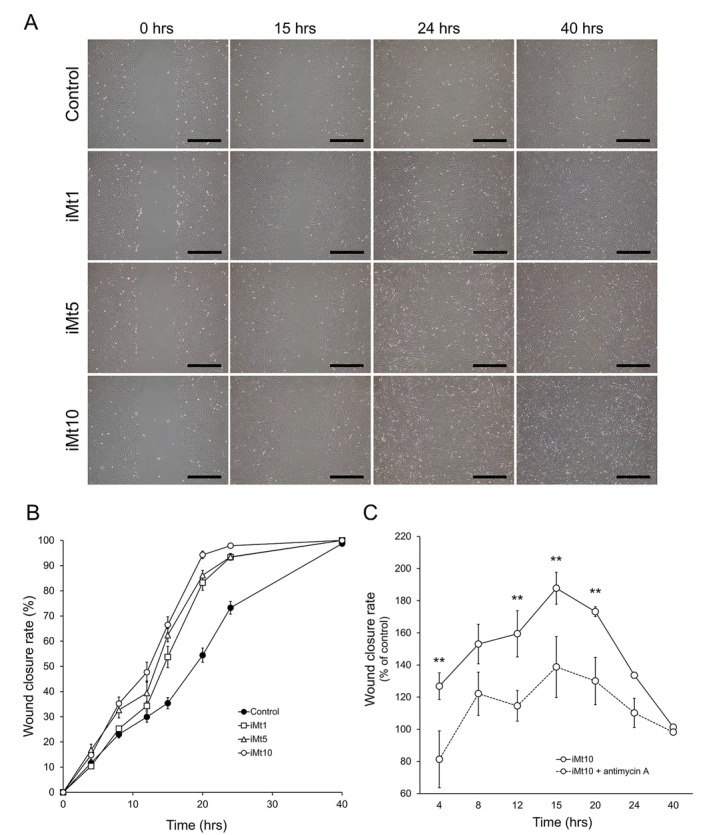
Scratch wound healing assay in vitro following isolated mitochondria (iMt) transplantation from normal human dermal fibroblasts (NHDF). (A) Microscopic images of scratch wound healing assay for each iMt concentration. Scale bar = 1 mm. (B) Wound closure rate for each iMt concentration. iMt1, iMt5, and iMt10 indicate administration of isolated mitochondria at concentrations of 1, 5, and 10 μg/cm^2^, respectively. Data represent the mean ± standard error of the mean. Statistical analysis was performed using two‐way analysis of variance (ANOVA) followed by Dunnett's multiple comparisons test versus the control group at each time point (*n* = 6 for each condition). (C) Comparison of wound closure rate for iMt10 with and without the mitochondrial function inhibitor antimycin A. Data represent the mean ± standard error of the mean. Statistical analysis was performed using two‐way ANOVA followed by Sidak's multiple comparisons test (**p < 0.01; *n* = 6 for each condition).

The scratch wound healing assay was performed for investigating the effects of iMt administration to NHDF on wound healing. All of the NHDF groups with transplanted iMt covered the scratched wound faster compared with the control group, the effect of which increased in a dose‐dependent manner (Figure 2A,B). Two‐way analysis of variance (ANOVA) demonstrated significant effects of treatment group (*p* < 0.001), time (*p* < 0.001), and treatment‐by‐time interaction (*p* < 0.001). Dunnett's multiple comparisons test revealed significantly greater wound closure in all iMt‐treated groups compared with controls (all *p* < 0.001). The wound closure rate of iMt10, which yielded the highest results in the scratch wound healing assay, demonstrated a wound closure rate of up to 1.8 times higher than controls. Two‐way ANOVA demonstrated significant effects of treatment group (*p* < 0.001) and time (*p* < 0.001), whereas no significant treatment‐by‐time interaction was observed (*p* = 0.385). This effect was attenuated by treatment with antimycin A (Figure 2C). The above findings suggested that the administration of iMt could accelerate wound closure in vitro.

**FIGURE 3:**
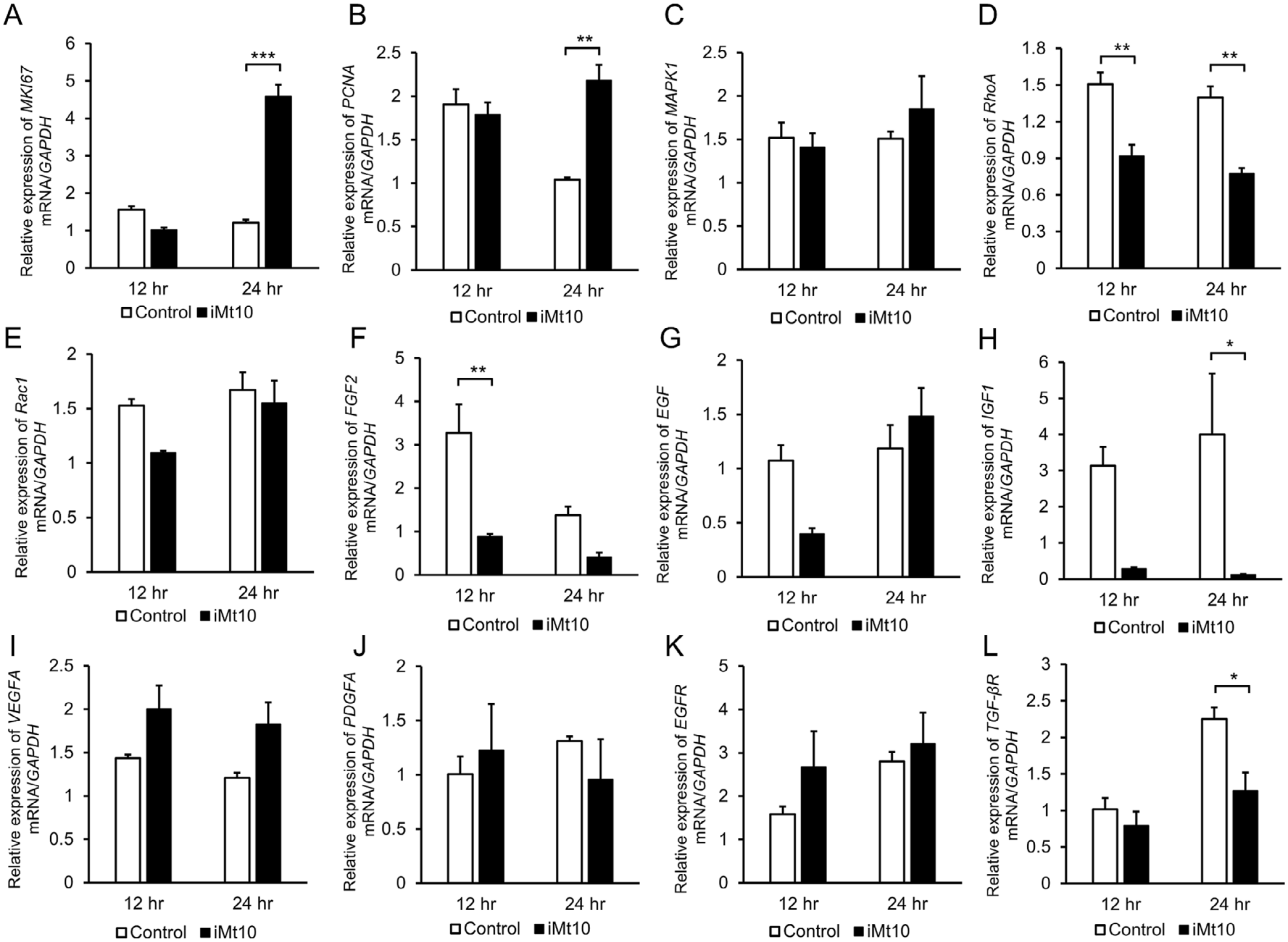
Gene expression analysis after treatment with isolated mitochondria (iMt) into normal human dermal fibroblasts (NHDF). Gene expression of markers for cell proliferation (A: *MKI67*, B: *PCNA*, C: *MAPK1*), cell migration ability (D: *RhoA*, E: *Rac1*), growth factors (F: *FGF2*, G: *EGF*, H: *IGF1*, I: *VEGFA*, J: *PDGFA*), and membrane receptors (K: *EGFR*, L: *TGF‐βR*) of NHDF was analysed 12 and 24 h after iMt administration in the scratch wound healing assay. Data represent the mean ± standard error of the mean. Statistical analysis was performed using two‐way analysis of variance followed by Sidak's multiple comparisons test (*p < 0.05, **p < 0.01, ***p < 0.001; *n* = 3 for each condition).

**FIGURE 4:**
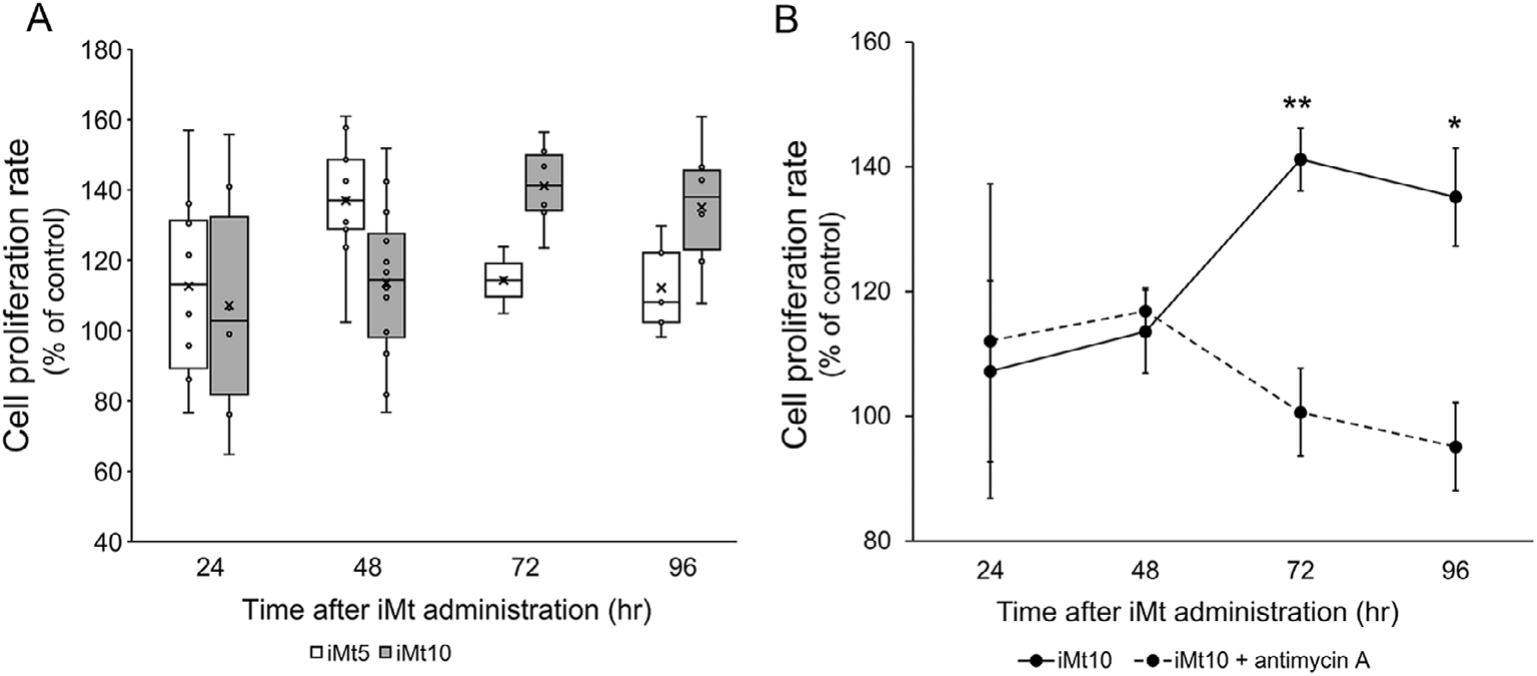
Cell proliferation assay after treatment with isolated mitochondria (iMt) in normal human dermal fibroblasts (NHDF). (A) Cell proliferation rate was measured over time after administration of iMt5 or iMt10 to NHDF in the logarithmic phase. Values were normalised to the corresponding control group at each time point. Data represent the mean ± standard error of the mean. Statistical analysis was performed using two‐way analysis of variance (ANOVA) followed by Sidak's multiple comparisons test (*n* = 3–12 per group depending on the time point). (B) Comparison of the cell proliferation rate between iMt10 and iMt10 treated with the mitochondrial function inhibitor antimycin A. Values were normalised to the corresponding control group at each time point. Data represent the mean ± standard error of the mean. Statistical analysis was performed using two‐way ANOVA followed by Sidak's multiple comparisons test (*p < 0.05, **p < 0.01; *n* = 3–12 per group depending on the time point).

In the scratch wound healing assay, gene expression analysis using the quantitative reverse transcription polymerase chain reaction was performed to investigate the cause of the early wound closure in the iMt groups. We observed no significant increase in the expression of cell proliferation‐related genes (*MKI67*, *PCNA*, and *MAPK1*) at 12 h after iMt administration (Figure 3A–C). However, *MKI67* and *PCNA* expression were significantly increased at 24 h after treatment (*p* < 0.001 and *p* = 0.002, respectively), whereas *MAPK1* expression was not significantly altered. Two‐way ANOVA demonstrated significant effects of treatment group, time, and treatment‐by‐time interaction for *MKI67* (all *p* < 0.001), and significant effects of treatment group (*p* = 0.019) and treatment‐by‐time interaction (*p* = 0.007) for *PCNA*. Among migration‐related genes, *Rac1* expression was not significantly altered at either time point, whereas *RhoA* expression was significantly decreased following iMt administration at both 12 and 24 h (*p* = 0.002 and *p* = 0.001, respectively) (Figure 3D,E). No significant changes were observed in the expression of *EGF*, *VEGFA*, *PDGFA*, or *EGFR* following iMt administration (Figure 3G,I–K). In contrast, *FGF2* expression was significantly decreased at 12 h (*p* = 0.004), whereas *IGF1* and *TGF‐βR3* expression were significantly decreased at 24 h (*p* = 0.035 and *p* = 0.018, respectively) (Figure 3F,H,L). However, these changes were limited to individual genes and time points, and no consistent pattern was observed among growth factor‐ or receptor‐related genes. Taken together with the increased expression of *MKI67* and *PCNA*, these findings suggest that enhanced cell proliferation contributes to the accelerated wound closure induced by iMt administration. Therefore, we performed a cell proliferation assay next. When iMt were administered during the exponential growth phase of NHDF, cell proliferation rate was approximately 140% at maximum compared with the control group (Figure 4A). Similar rates were observed for iMt5 and iMt10. Two‐way ANOVA revealed a significant interaction between treatment dose and time (*p* = 0.048), whereas no significant main effects of treatment dose (*p* = 0.363) or time (*p* = 0.153) were detected. This effect lasted 3–4 days and was inhibited by treatment with antimycin A (Figure 4B). Two‐way ANOVA demonstrated significant effects of treatment group (*p* = 0.020) and treatment‐by‐time interaction (*p* = 0.035).

**FIGURE 5:**
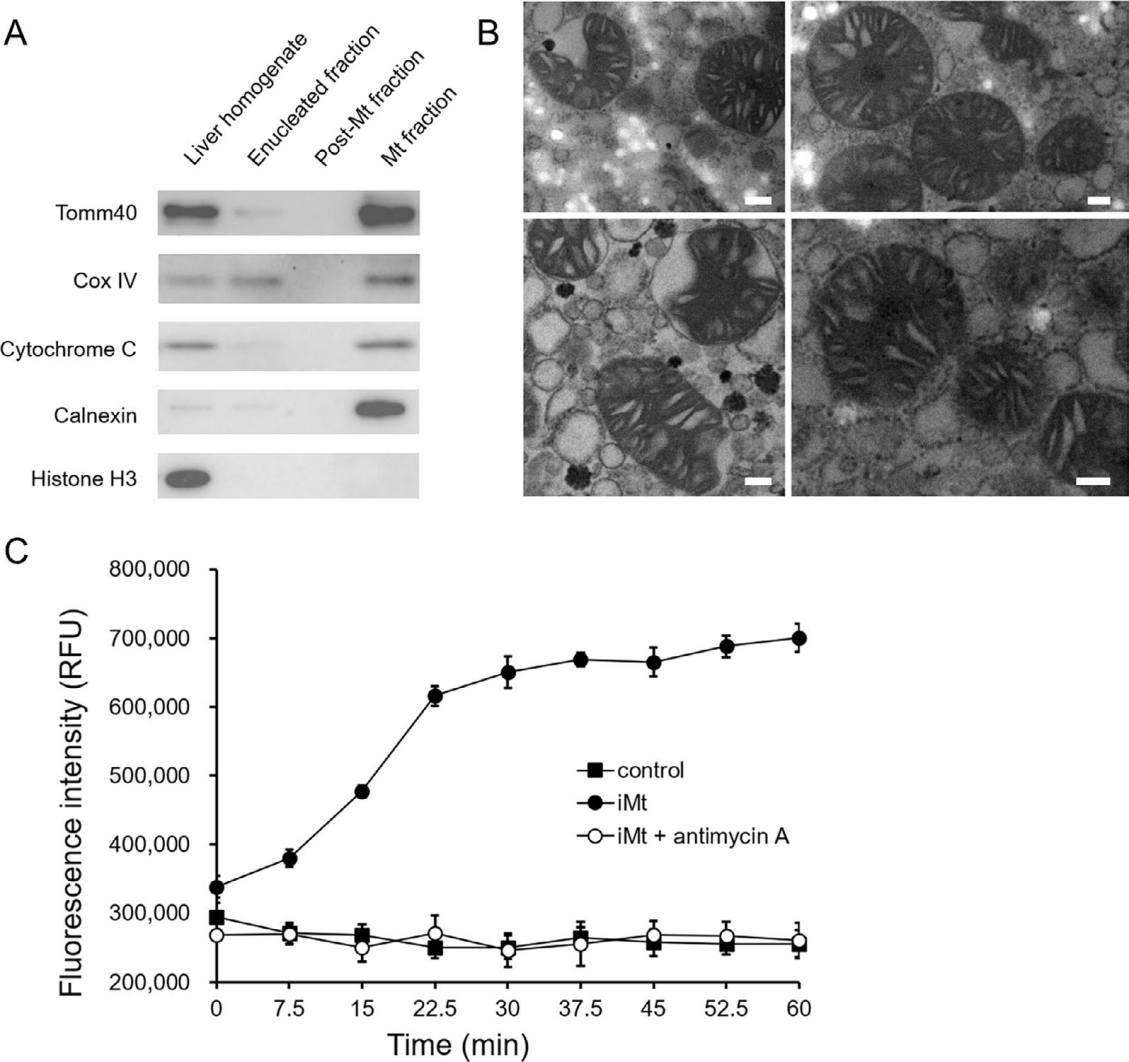
Isolation and functional evaluation of mouse liver‐derived mitochondria. (A) Western blot of each fraction obtained from murine liver homogenates. (B) Electron microscopy of isolated mitochondria (iMt) in the mitochondrial fraction. Scale bar = 200 nm. (C) Oxygen consumption rate assay using iMt with and without the mitochondrial function inhibitor antimycin A. Data represent the mean ± standard error of the mean (*n* = 3 for each condition).

**FIGURE 6:**
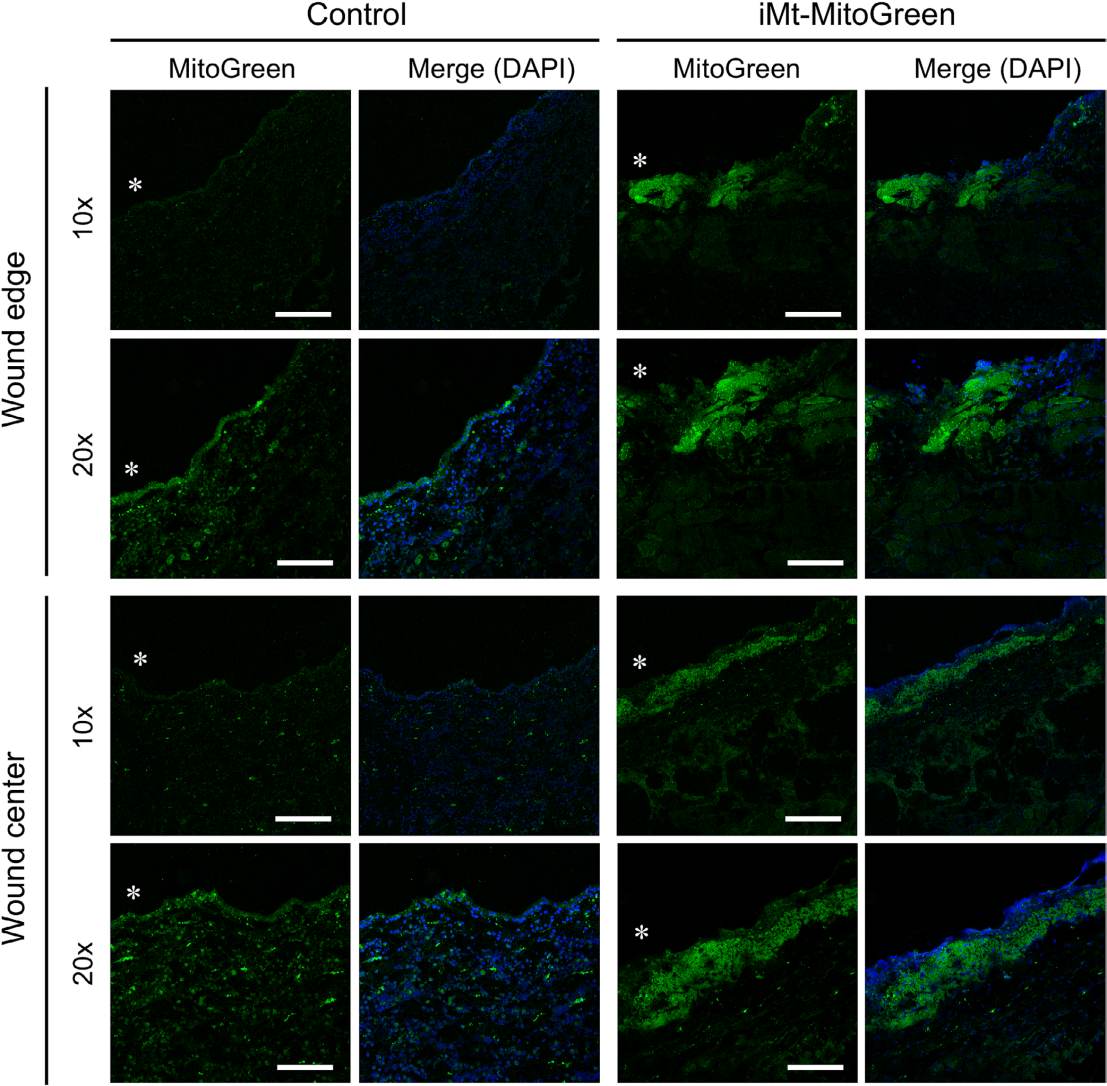
In vivo uptake of fluorescence‐labelled mouse liver‐derived mitochondria in wound tissues. Representative fluorescence images of wound tissues collected 24 h after treatment with fluorescently labelled isolated mitochondria (iMt‐MitoGreen). MitoGreen‐derived fluorescence signals were observed in both the wound edge and wound center of the iMt‐MitoGreen group and were more prominent than those in the control group. Nuclei were counterstained with DAPI (blue). Merged images are shown in the right panels. Asterisks indicate the wound surface. Scale bars = 200 μm (10×) and 100 μm (20×).

**FIGURE 7:**
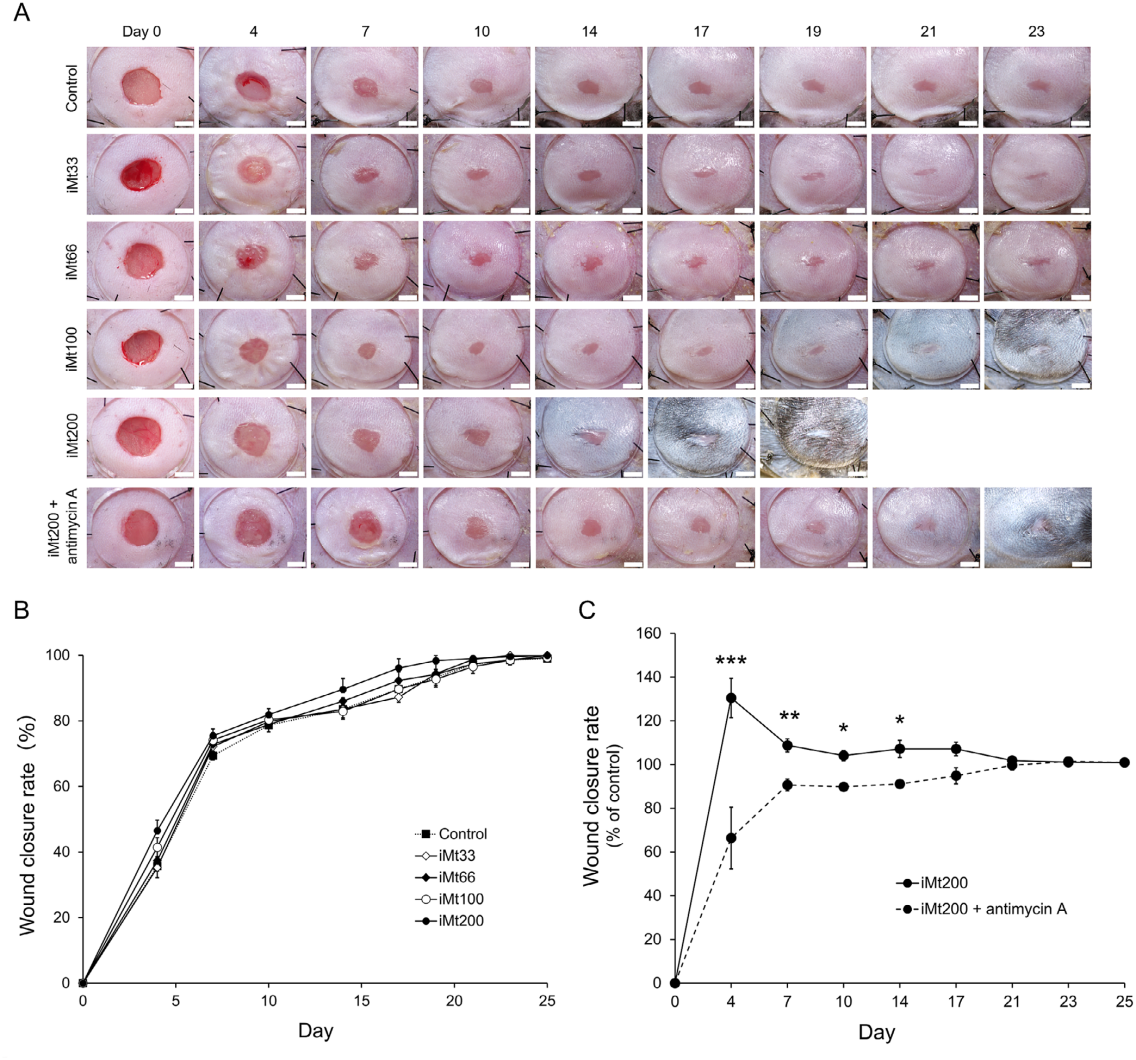
In vivo wound healing assay at each concentration of isolated mitochondria (iMt) derived from mouse livers. (A) Wound images after administration of iMt derived from mouse livers. Scale bar = 2 mm. (B) Wound closure rate at each iMt concentration. Data represent the mean ± standard error of the mean. Statistical analysis was performed using two‐way analysis of variance (ANOVA) followed by Dunnett's multiple comparisons test (*n* = 5–8 per group depending on the time point). (C) Comparison of wound closure rate for iMt200 with and without the mitochondrial function inhibitor antimycin A. Data represent the mean ± standard error of the mean. Statistical analysis was performed using two‐way ANOVA followed by Sidak's multiple comparisons test (*p < 0.05, **p < 0.01, ***p < 0.001; *n* = 6 for each condition).

The mouse wound healing model was used to assess the effects of iMt treatment in vivo on wound healing. For the in vivo experiments, mitochondria were isolated from the livers of allogenic mice, which provide a high and reproducible mitochondrial yield, following confirmation of morphology and function (Figure 5A–C). To investigate the in vivo distribution of administered mitochondria, iMt were labelled with MitoGreen and applied to wounds immediately after wound creation. Twenty‐four hours later, MitoGreen‐derived fluorescence signals were detected in both the wound edge and wound center of the treated group (Figure 6). These signals were more prominent than those observed in control wounds. The presence of MitoGreen‐derived fluorescence signals within wound tissues suggests that mitochondria‐derived signals reached and persisted in the wound environment for at least 24 h after treatment. As in the in vivo assays, wound closure was faster in the iMt‐treated group than in the control group, for which we observed a dose‐dependent effect (Figure 7A,B). Two‐way ANOVA demonstrated significant effects of treatment group (*p* < 0.001) and time (*p* < 0.001), whereas no significant interaction between treatment and time was observed (*p* = 0.712). The greatest effect was achieved by iMt200 at up to 140% wound closure compared with controls at 4 days after treatment, with a decreasing effect thereafter (Figure 7C). Two‐way ANOVA demonstrated significant effects of treatment group (*p* < 0.001) and treatment‐by‐time interaction (*p* < 0.001), whereas no significant effect of time was observed (*p* = 0.982). This result was prevented by antimycin A treatment.

## 4 Discussion

Previous reports [26, 27, 28, 29, 30, 31, 32] have demonstrated mitochondrial transplantation as a useful therapeutic approach for cells and tissues with dysfunctional mitochondria. In contrast, the present study investigated the effect of mitochondrial transplantation on wound healing in cells and tissues with normal mitochondria that have sustained damage, such as in acute wounds. In vitro, mitochondrial transfer accelerated wound closure and was associated with increased cell proliferation, while in vivo, it accelerated wound closure in a dose‐dependent manner.

Three pathways are known for the import of mitochondria into cells [37]: transient cellular connections known as tunnelling nanotubes (TNTs) [38, 39], extracellular vesicles (EVs) [40, 41], and the direct uptake of mitochondria released from cells [22, 42, 43]. This investigation employed a method involving the third transport pathway, which did not require special mechanisms such as TNTs or the preparation of new vesicles for EVs, but rather captured iMt by a phagocytic mechanism suggestive of micropinocytosis [44] simply by co‐culture with target cells or direct application onto the wound surface. However, additional research is required as no reports have compared the above three methods regarding iMt uptake efficiency.

Generally, cell proliferation requires the synthesis of biomolecules such as DNA, proteins, and lipids [45, 46], along with cell cycle checkpoint mechanisms [47] and mitosis [48]. Mitochondria are considered a crucial source of ATP for these processes [35]. In this study, although treatment with iMt did not significantly alter the migration ability of NHDF, it markedly increased their proliferative capacity. Guo et al. [49] reported similar findings, demonstrating enhanced proliferation and migration following administration of mitochondria derived from bone marrow mesenchymal stem cells. The mechanisms by which mitochondrial administration preferentially enhanced proliferation rather than migration in the present study remain unclear. Further investigation will be required to determine the molecular mechanisms underlying these differential cellular responses.

To examine the fate of administered mitochondria in vivo, iMt were labelled with MitoGreen and tracked within wound tissues. MitoGreen‐derived fluorescence signals were detected in wound sections 24 h after treatment, indicating that administered mitochondria reached and remained within the wound environment. Although the present study did not identify the precise cellular localization of the administered mitochondria, these findings support the possibility that administered mitochondria are present within the wound environment and contribute to the observed enhancement of wound healing. In the in vivo experiments, mitochondria were isolated from mouse liver rather than dermal fibroblasts. Liver tissue was selected because it provides a high and reproducible mitochondrial yield from a single donor animal. In our experimental setting, approximately 3–5 mg of mitochondrial protein could be obtained from a single mouse liver, which was sufficient for administration in multiple wounds. In contrast, obtaining comparable quantities of mitochondria from primary dermal fibroblasts would require extensive cell expansion. Such prolonged culture may alter both cellular characteristics and mitochondrial function. Therefore, liver‐derived mitochondria were used as a practical and reproducible source for in vivo administration. Nevertheless, whether the tissue origin of donor mitochondria influences therapeutic efficacy remains an important question that warrants future investigation.

Growth factors and cytokines that promote cell proliferation, including FGF2, EGF, IGF‐1, PDGF, VEGF, and TGF‐β, are released from NHDF in an autocrine and paracrine manner [50, 51, 52, 53]. This study employed differential centrifugation to obtain the mitochondrial fraction, which also contained organelles and other substances. It was therefore possible that growth factors and cytokines promoted cell proliferation, although gene expression analysis of each factor and its receptor did not exhibit a significant increase over the control group. Thus, the contribution of contaminating growth factors or cytokines appeared limited under the conditions examined. As treatment of this fraction with a mitochondrial function inhibitor inhibited the increases in cell proliferation rate both in vitro and in vivo, we considered that the findings obtained were primarily mediated by iMt.

Although the present study demonstrated beneficial effects of mitochondrial transplantation in an acute wound model, mitochondrial dysfunction was not directly evaluated in either the in vitro or in vivo experimental settings. Therefore, the observed therapeutic effects cannot be attributed to the correction of pre‐existing mitochondrial dysfunction. Mitochondrial dysfunction has been implicated in chronic wounds, including diabetic ulcers, where impaired bioenergetics and oxidative stress contribute to delayed healing. Therefore, mitochondrial transplantation may exert greater therapeutic effects under such pathological conditions. Future studies using diabetic or chronic wound models will be necessary to further define the therapeutic potential and mechanisms of mitochondrial transplantation.

Several study limitations should be acknowledged. First, although MitoGreen‐derived fluorescence signals were detected within wound tissues and administered mitochondria were confirmed to be taken up by cultured fibroblasts, the specific cell populations responsible for mitochondrial uptake within wound tissues were not identified. Future studies using cell type‐specific markers will be required to clarify the cellular targets of mitochondrial transplantation in vivo. Second, while mitochondrial transplantation enhanced fibroblast proliferation and accelerated wound closure, we did not directly assess ATP production, mitochondrial content, or bioenergetic activity within wound tissues. Third, reactive oxygen species generation and other downstream mitochondrial signalling pathways were not evaluated. These analyses may provide further insight into the mechanisms by which administered mitochondria promote wound repair. Fourth, the in vivo wound healing response was primarily evaluated by macroscopic wound closure, and detailed histological assessments, including re‐epithelialization, granulation tissue formation, and cellular proliferation within wound tissues, were not performed. Finally, mitochondria used for the in vivo experiments were isolated from mouse liver because this tissue provides a high and reproducible mitochondrial yield. However, whether the tissue origin of donor mitochondria influences therapeutic efficacy remains unclear and warrants further investigation. Future studies incorporating histological, metabolic, and cell type‐specific analyses will help clarify the mechanisms underlying mitochondrial transplantation‐mediated wound healing.

## 5 Conclusions

We demonstrated that administration of isolated mitochondria to NHDF enhanced cell proliferation without significantly affecting cell migration in vitro. In addition, mitochondrial transplantation accelerated acute wound closure in a mouse wound healing model. Furthermore, MitoGreen‐derived fluorescence signals were detected within wound tissues 24 h after treatment, supporting the presence of mitochondria‐derived fluorescence signals within the wound environment. These findings may provide key insights into novel therapeutic approaches for wound healing.

## Author Contributions

Ikkei Takashimizu designed and performed all experiments and wrote this manuscript. Miho Noguchi contributed to the Western blot analysis. Shunsuke Yuzuriha directed the investigation. All authors have read and approved the final manuscript.

## Funding

This work was supported by the Japan Society for the Promotion of Science (JSPS) KAKENHI (Grant Numbers JP18K16982 and JP24K12844).

## Conflicts of Interest

The authors declare no conflicts of interest.

## Data Availability

All data generated or analysed during this study are included in this published article.
